# Elderly primary care hypertension patients–who to refer for echocardiography?

**DOI:** 10.1007/s12471-014-0550-z

**Published:** 2014-03-28

**Authors:** S. C. A. M. Bekkers, H. P. Brunner-La Rocca

**Affiliations:** Department of Cardiology, Maastricht University Medical Center, P. Debyelaan 25, PO Box 5800, 6202 AZ Maastricht, the Netherlands

Heart failure (HF) is a complex clinical syndrome resulting from impaired diastolic and/or systolic function and clinically manifested by numerous, rather unspecific symptoms such as dyspnoea (at rest or exertion), orthopnoea, wheezing, chronic fatigue and lower extremity oedema. The prevalence of HF increases steeply with age, causing high mortality and morbidity, substantial loss in quality of life, and high health care costs [1]. For the diagnosis of HF, guidelines require, in addition to symptoms, objective evidence of cardiac dysfunction that is most commonly assessed by echocardiography. While many conditions may cause HF, the most common aetiologies are coronary artery disease (CAD) and hypertension.

Early diagnosis and adequate treatment are necessary to improve symptoms and prognosis. General practitioners (GPs) and nurse practitioners (NPs) play a key role in identifying HF, but diagnosing HF in primary care remains challenging because symptoms are often unspecific, subtle or even absent [2]. Advanced diagnostics are often not easily available, but diagnostic uncertainty may lead to inappropriate treatment of wrongly diagnosed HF or delaying therapy when the diagnosis is missed.

Echocardiography is the reference investigation for left ventricular dysfunction and plays an important role to diagnose both diastolic and systolic dysfunction of the left ventricle (class I, level C) [3]. There is an increase in the number of open access echocardiography services to primary care for early diagnosis and appropriate treatment of cardiac dysfunction and HF. However, due to limited resources, adequate screening by GPs or NPs remains imperative for cost-effective use of limited resources. The question, therefore, arises which patients should be investigated by echocardiography to provide best care. The equivocal recommendation by the most recent guidelines on the management of arterial hypertension by the ESC underscores the difficulty of this question [4].

In this issue of the Netherlands Heart Journal, Ringoir et al. report on the prevalence of symptoms related to HF in 591 elderly primary care patients with hypertension and the diagnostic value of these symptoms to predict cardiac dysfunction, defined as an abnormal echocardiogram [5]. The investigators included both systolic (LVEF < 55 %) and diastolic dysfunction parameters (left atrial volume index > 29 ml/m^2^, E/a ratio < 1 and DT > 200 ms in presence of left ventricular hypertrophy), as well as valvular abnormalities, wall motion abnormalities, and right ventricular hypertrophy (≥ 6 mm) in their definition of an abnormal echocardiogram, which was found in 30 % of patients. Symptoms (i.e. restless sleep, cold extremities, fatigue, shortness of breath, and ankle oedema) were present in 13–25 % of the study population. Although these symptoms were not sensitive (20–32 %), they were rather specific for an abnormal echocardiogram (71–90 %). Of these, the most specific symptom (i.e. ankle oedema) was, however, also the least sensitive one. Still, it was the only one adding to the risk of cardiac dysfunction independently to already known clinical risk factors. The authors concluded that elderly hypertensive primary care patients presenting with ankle oedema might be appropriately referred for echocardiographic screening. The question is if this statement is correct and how it should be put into clinical perspective.

The investigators are to be complimented with their study, because it addresses an important topic with many unresolved issues, especially in primary care patients. Their results add another missing piece to the diagnostic strategy puzzle in primary care patients with suspected HF. However, the conclusion from the results may be less positive than the authors suggested, but this is nonetheless important. Obviously, patients with clinical signs and/or symptoms possibly related to HF, particularly if alternative explanations are absent, should be referred for further diagnostics. This is reflected by the high specificity, resulting in an acceptable positive predictive value. However, this is clinically rather obvious and only of significant value if the same test (in this case signs or symptoms) is sufficiently sensitive with adequate negative predictive value to safely exclude a relevant disorder (in this case cardiac dysfunction). This is apparently not the case! Thus from a primary care perspective, the low sensitivity of this and other clinical signs does not allow reliable screening because the large majority of patients with cardiac dysfunction would still be missed.

The results of this study may be in some contrast with other studies. Thus, Roalfe et al. investigated the diagnostic role of clinical features and NT-proBNP. They identified four simple clinical features (Male, history of myocardial Infarction, lung Crepitations, and oEdema: so called MICE) that could accurately triage between patients with suspected HF who should be referred for echocardiography directly and those in whom referral should depend on NT-proBNP values (sensitivity 90–96 %, specificity 58–63 %) [6]. However, the population included in this analysis was at higher risk, which may have contributed to the better performance of clinical features for screening of HF.

In another large Dutch cross-sectional study, 721 primary care patients with almost similar baseline characteristics (age 71 ± 12 years, 65 % females, 52 % hypertension, 7 % CAD) suspected of having HF (final prevalence 29 %) underwent a standardised diagnostic work-up that included history, physical examination, ECG, X-ray, spirometry, laboratory testing, and echocardiography. Hierarchical multivariable logistic regression modelling revealed that the combination of three items from history (age, CAD, and loop diuretics) plus six from physical examination (pulse rate and irregularity, displaced apical beat, murmur, rales, and increased jugular venous pressure) predicted HF quite accurately (c-statistic 0.83). The addition of NT-proBNP to the diagnostic workup added diagnostic power and increased the c-statistic to 0.86. The diagnostic rule derived from this study showed to be very robust when used in external validation studies (c-statistic 0.88–0.95) [7]. This indicates that information obtained from the history and physical examination may be of importance if HF is suspected in relatively high-risk populations, but they may be considerably less valuable in patients at low risk for HF. Unfortunately, Ringoir et al. fail to give the prevalence of HF, but this is clearly much lower than the prevalence of echocardiographic abnormalities.

Another important shortcoming is the fact that ECGs were not part of the focus of their study. However, hypertensive heart disease is often associated with electrocardiographic (ECG) abnormalities and a 12-lead ECG is recommended in *all* patients for screening of cardiac dysfunction in hypertension [3, 4]. Although there are clear limitations, an abnormal ECG can detect echocardiographic left ventricular systolic dysfunction in suspected HF patients (area under the summary ROC curve 0.84, 95 % CI: 0.33–1). Conversely, a normal ECG recording may exclude left ventricular systolic dysfunction in most cases [8]. Therefore, the diagnostic value of signs and/or symptoms of HF as a rather isolated screening tool is clinically less relevant. Moreover, the authors do not provide a model of the diagnostic accuracy by combining all clinical parameters found to be significantly correlated with the presence of cardiac dysfunction. Additionally, laboratory test including NT-proBNP measurements could have significantly increased diagnostic yield, but such information is lacking.

One important reason for the discrepancies between this study and others may be related to differences in defining HF. A uniform definition of HF is well published in the guidelines, but in clinical reality and also in some of the studies mentioned, different definitions were used, making direct comparison difficult. Moreover, the investigators of the current study used different cut-off values to define systolic dysfunction than proposed by international guidelines (ESC: LVEF < 50 %) [3]. Similarly, diastolic dysfunction was not defined according to current recommendations (LA volume index > 34 mL/m^2^, E/a > 1 [pseudonormal or restrictive pattern], DT < 150 ms, S/D < 1, e’ < 8 cm/s, E/e’ >15, etc.) [9]. Even more importantly, the authors do not provide separate analysis for patients with reduced and those with preserved ejection fraction. Although we agree with the authors that investigating only systolic dysfunction is missing an important group of patients with clinically relevant cardiac dysfunction, the distinction is very relevant as treatment differs significantly between the two. In fact, one reason for performing echocardiography may be the distinction between preserved and reduced left ventricular systolic function. Whereas the former is important for risk stratification and more aggressive treatment of risk factors, the latter requires additional specific treatment even if asymptomatic [3].

The results of this study do emphasise that diagnosing cardiac dysfunction in primary care remains difficult and cannot be reliably performed based on clinical signs and symptoms alone. Previous studies suggest that the combination of clinical signs, patient characteristics and additional testing such as ECG and NT-proBNP may be more accurate, at least in screening patients with suspected HF in primary care and triage patients for echocardiography. However, prospective studies should validate this, as is currently done in the REFER (REFer for EchocaRdiogram) study [10]. Moreover, similar studies in lower risk patients as investigated by Ringoir et al. are urgently needed. Until such results become available, we recommend following the guidelines for management of arterial hypertension [4]: all patients with hypertension should receive an ECG in addition to the clinical evaluation. Those with any abnormal finding or high risk of having heart failure should be referred for echocardiography.
